# Supplementary material for: Super-Resolution Microscopy Reveals a Direct Interaction of Intracellular Mycobacterium tuberculosis with the Antimicrobial Peptide LL-37
Source: Int J Mol Sci. 2020 Sep 14;21(18):6741. doi: 10.3390/ijms21186741 (PMC7555347; doi:10.3390/ijms21186741)
Supplement: Supplementary file 1 [file ijms-21-06741-s001.pdf]

Supplementary Materials:

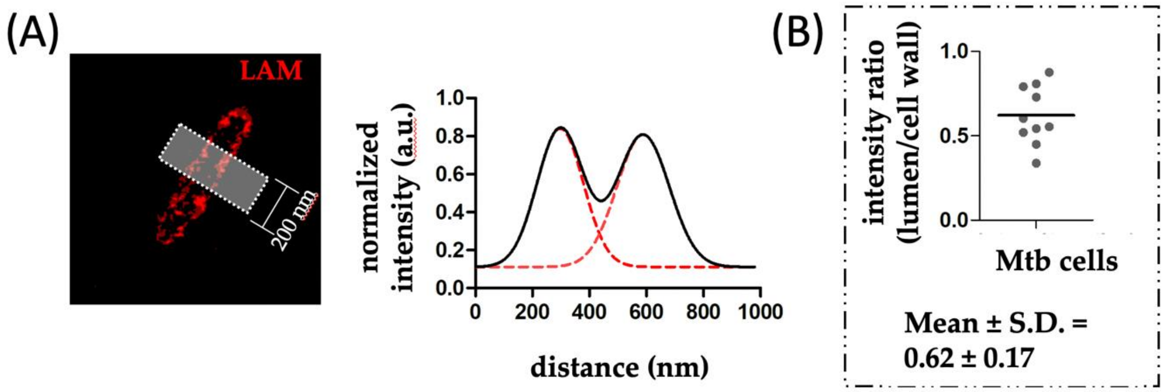

Figure S1. Cell wall specific labelling with LAM antibody determined by STED microscopy. (A) Image of an extracellular *Mtb* immune-labelled for LAM. A 200nm wide cross-section is used to quantify the fluorescence intensity at the cell perimeter compared to the cell interior. (B) Statistical analysis of cross-sections taken from ten different *Mtb* cells.

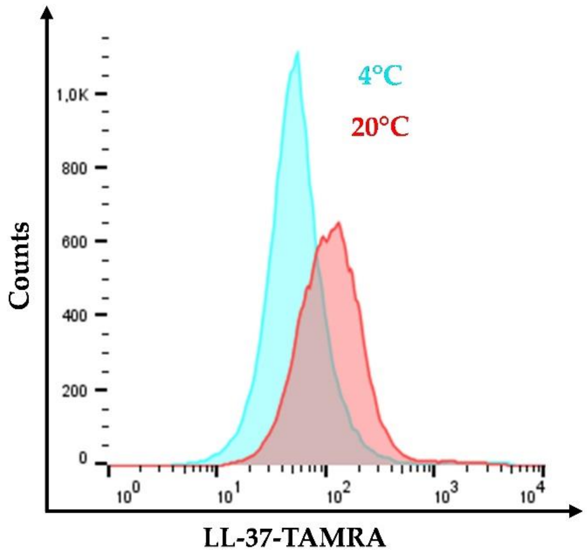

Figure S2. Effect of temperature on the internalization of LL-37-TAMRA. Macrophages were incubated with LL-37-TAMRA (11µM) at 4 °C or 20 °C. The histogram shows the mean fluorescence intensity for TAMRA at 4 °C (blue) and 20 °C (red) determined by FACS analysis. Representative histogram of five donors with similar result is shown.

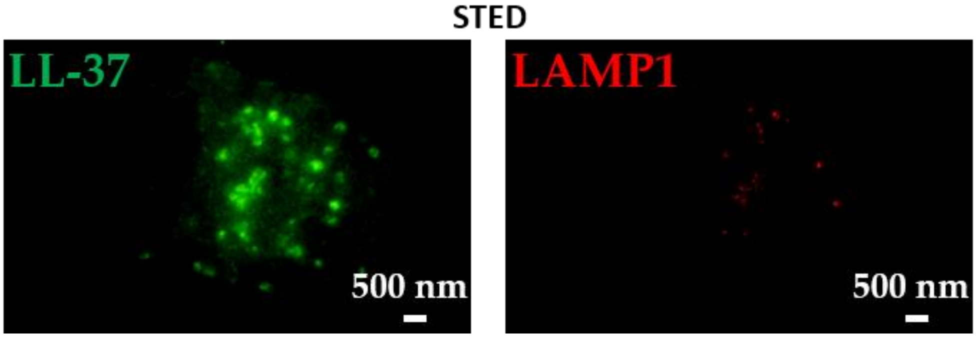

Figure S3. Phagolysosome in un-infected macrophages after incubation with LL-37-TAMRA. Un-infected macrophages were incubated with LL-37-TAMRA for 30 min. Lysosomes were labelled using an anti-LAMP1 antibody. Representative area of three different donors is shown. LL-37-TAMRA is depicted in green, lysosomes in red. Images were acquired using dual color STED microscopy. Scale bar is 500 nm.

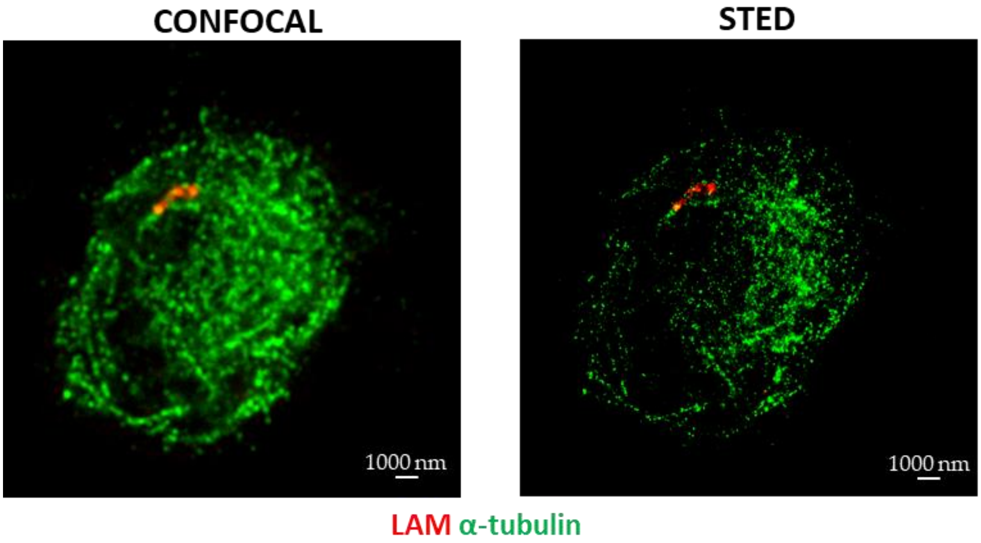

Figure S4. Visualizing intracellular *Mtb.* Macrophages were infected with Mtb. The microtubule network of the macrophage cell was visualized with α-tubulin labelling while internalized bacterial cells were labeled using an anti-LAM-antibody. Representative area from three different donors is shown. Mtb are depicted via lipoarabinomannan (LAM) in red, α-tubulin in green. Images were acquired first with confocal technique (left) and then using dual color STED (right) microscopy. Scale bars represent 1000 nm.

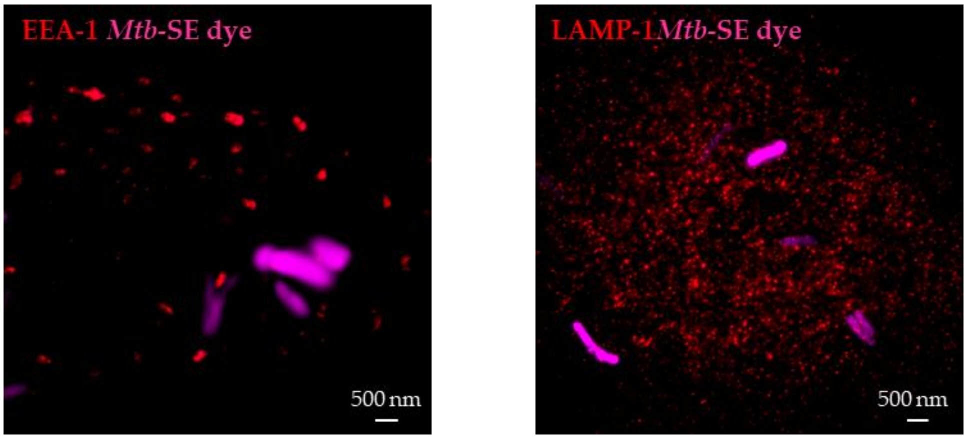

Figure S5. Internal *Mtb* does not co-localize with EEA1 or LAMP-1 in absence of LL-37. Macrophages were infected with *Mtb* labelled with fluorescein succinimidyl ester Atto 647N. Early endosomes were detected using an anti-EEA1 antibody (A) and phagolysosome using LAMP-1 (B). *Mtb* are depicted in magenta while early endosomes (A) and LAMP-1 labelling (B) is in red**.** Images were acquired using dual color STED microscopy. Scale bar represents 500 nm.

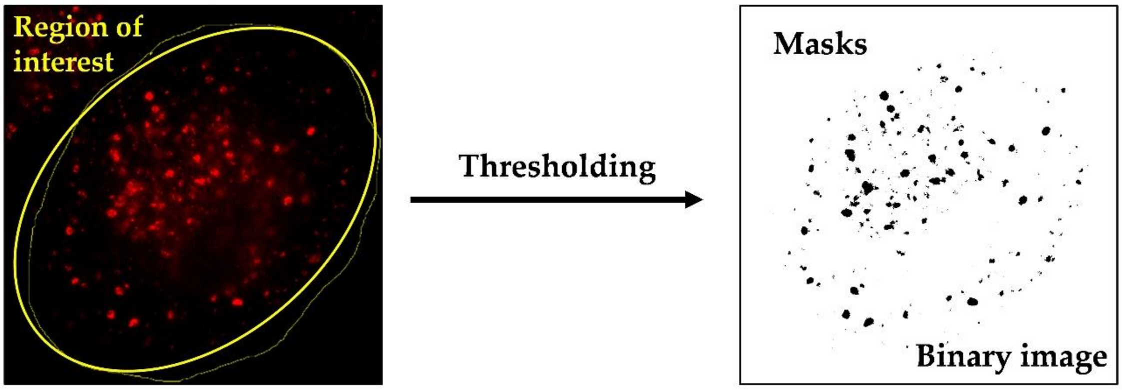

Figure S6. Image analysis to quantify perimeter of early endosomes. The image shows a representative single macrophage with the region of interest depicted in yellow. Image analysis was performed using Image J (v1.52c), including the add-on tool „Analyze Particles“. This procedure excludes pixels below an intensity threshold and converts the remaining pixels into a binary mask image which are then analysed. Final quantification of endosomal perimeter was then performed via GraphPad Prism (Version 8.2.1).
